# Taking Impressions and Fitting Artificial Dentures

**Published:** 1897-02

**Authors:** W. T. Magill

**Affiliations:** Rock Island, Ill.


ARTICLE II.

### TAKING IMPRESSIONS AND FITTING ARTIFICIAL DENTURES.

DR. W. T. MAGILL, ROCK ISLAND, ILL.

First provide every article that will be needed in taking an impression. A good supply of impression trays or cups of various shapes and sizes should be at hand to choose from. The cup should be as nearly as possible the size and shape of the alveolar ridge. If an upper impression is to be taken, seat your patient in the operating chair in a nearly upright position, tipped a little back. Spread a large, white, clean napkin over and in front of the patient. Fasten it around his neck to prevent clothing from being soiled with water, wax, plaster, or any other material you may use. Now examine carefully the mouth and jaw, noting any peculiarities, protuberances, undercuts, hard and soft points, feeling with the index finger to ascertain how much the soft parts yield to and the hard resist pressure. The soft parts will need compression in proportion to the amount of yielding, and the hard parts will need easing in proportion to the amount of resistance. Select an impression tray of proper size and form. Everything being in readiness, we take our modeling compound, or yellow wax, from the clean hot water, when sufficiently soft to work up into a form to correspond with the size and form of tray to be used. Fill the tray a little above the top of the rim, leaving the surface of the wax or modeling compound smooth without any folds or creases; rewarm the surface over a spirit lamp, Bunsen burner, or by dipping it in hot water. Then quickly, dexteriously and skilfully place it in the mouth, by "placing the heel of the tray in against the right corner of the mouth, and with the index finger of the left hand stretch the left corner of the mouth till the tray slips in." Now with a quick movement of the tray held in the right hand, press steadily and firmly upward and slightly backward, at the same time being careful to observe that the mucous membrane is not folded in between the impression composition and alveolar ridge, which sometimes occurs, particularly in taking impressions of the lower jaw. This can be overcome by distending the cheek with the index finger of either hand placed well back in the mouth and pressing outward, keeping the pressure on the tray steady, avoiding all rocking motion. To hasten the operation at this point have an assistant inject with a syringe a little ice cold water around the margin of the tray, tipping the head of the patient a little forword to avoid choking. When sufficiently hardened, remove by gently working the tray in a somewhat rotary motion with the right hand, and distending the cheek and muscles with the index finger of the left hand till the atmospheric pressure is relieved, then remove in the same manner that it was inserted, being careful not to drag, bend or break by using too much force, or being too hasty.

A perfect impression can be relied on in proportion to the amount of resistance required to overcome the atmospheric pressure. An impression which does not adhere firmly to the roof of the mouth or alveolar ridge, cannot be relied on to give satisfactory results, while fairly good results can be obtained from impressions taken in wax, gutta-percha, modeling compound and plaster of Paris alone. The following process is recommended for a more perfect impression:

Take an impression in wax or modeling compound, preferably the latter, trim off the surplus of overhanging portions, warm the margin all around and press outward about a line; the heel or posterior part of the impression should be pressed or turned up as much as three lines, while this part is still warm and soft. Should it become hard or unyielding, immerse the posterior palatal portion in hot water to soften. Now mix plaster of Paris of best quality (sifted) in a gill of warm water, in which 10 grains of salt has been dissolved, to the consistency of thick cream; fill the impression about half full, being careful to have every part of the surface covered with plaster; reinsert it in the mouth as above described; press firmly into position without rocking; hold till the plaster that remains in the rubber bowl will crack or break without crushing, then remove as before directed. Should there be any protuberances or undercuts the plaster (being very thin) will break or crack in withdrawing, and can be replaced and form a perfect impression that can be fully relied on for all practical purposes. Should there be any imperfections in the plaster, air bubbles or faulty places, chip out the plaster and refill as before and try again.

In following this method a minimum amount of plaster is used, and seldom if any overflows into the mouth, consequently no retching or irritating of the soft palate is produced, as is often the case when plaster alone is used. Salt is used for a dual purpose, viz., to hasten the setting of the plaster, and make it more brittle, a valuable quality where undercuts occur, or a partial impression is being taken; also, it diminishes the expansion of the plaster. After the plaster is sufficiently dried it should be stained with a thin solution of shellac varnish. When dry, immerse in a bowl of soapy water for five or ten minutes, then fill immediately, and there will be no sticking of cast to impression. Some operators simply immerse the impression in clear water without staining. Where the ruga are deep or there are undercuts, staining is valuable in determining where the line of separation is. "Plaster stirred longer than is required to thoroughly saturate it with water or where an extra quantity of water is used, the expansion is correspondingly greater," hence this often accounts for many rocking misfitting plates.

It is not always possible to get sufficient cohesion in a lower plate to retain it in its position during the act of mastication. Of the many devices which have been resorted to, to assure better cohesion, some prove beneficial; many are worthless. Experience teaches that the greater the surface covered the plate will be more likely to serve the purpose for which it is designed. I remember to have read in one of the old journals in regard to fitting lower plates, "trim off all you think it will bear, and then just a little more, and your plate will be about right." Now, I would say, add on all you think it will bear and just a little more, and in very many cases you will be charmed with the result, though not in such manner as to chafe or cut the muscles of the cheek. Upper plates can often be materially improved by being formed in the same manner.—*Dental Review.*

